# Breast cancer outcome prediction with tumour tissue images and machine learning

**DOI:** 10.1007/s10549-019-05281-1

**Published:** 2019-05-22

**Authors:** Riku Turkki, Dmitrii Byckhov, Mikael Lundin, Jorma Isola, Stig Nordling, Panu E. Kovanen, Clare Verrill, Karl von Smitten, Heikki Joensuu, Johan Lundin, Nina Linder

**Affiliations:** 1 Institute for Molecular Medicine Finland (FIMM), University of Helsinki, Helsinki, Finland; 2 Department of Cancer Biology, BioMediTech, University of Tampere, Tampere, Finland; 3 Department of Pathology, Medicum, University of Helsinki, Helsinki, Finland; 4 HUSLAB and Medicum, Helsinki University Hospital Cancer Center and University of Helsinki, Helsinki, Finland; 5 Nuffield Department of Surgical Sciences, University of Oxford, Oxford, UK; 6 NIHR Oxford Biomedical Research Centre, Oxford, UK; 7 Eira Hospital, Helsinki, Finland; 8 Department of Oncology, Helsinki University Hospital and University of Helsinki, Helsinki, Finland; 9 Department of Public Health Sciences, Karolinska Institutet, Stockholm, Sweden; 10 Department of Women’s and Children’s Health, International Maternal and Child health (IMCH), Uppsala University, Uppsala, Sweden; 11 Science for Life Laboratory (SciLifeLab), Karolinska Institutet, Solna, Sweden

**Keywords:** Breast cancer, Machine learning, Deep learning, Outcome prediction

## Abstract

**Purpose:**

Recent advances in machine learning have enabled better understanding of large and complex visual data. Here, we aim to investigate patient outcome prediction with a machine learning method using only an image of tumour sample as an input.

**Methods:**

Utilising tissue microarray (TMA) samples obtained from the primary tumour of patients (*N* = 1299) within a nationwide breast cancer series with long-term-follow-up, we train and validate a machine learning method for patient outcome prediction. The prediction is performed by classifying samples into low or high digital risk score (DRS) groups. The outcome classifier is trained using sample images of 868 patients and evaluated and compared with human expert classification in a test set of 431 patients.

**Results:**

In univariate survival analysis, the DRS classification resulted in a hazard ratio of 2.10 (95% CI 1.33–3.32, *p* = 0.001) for breast cancer-specific survival. The DRS classification remained as an independent predictor of breast cancer-specific survival in a multivariate Cox model with a hazard ratio of 2.04 (95% CI 1.20–3.44, *p* = 0.007). The accuracy (C-index) of the DRS grouping was 0.60 (95% CI 0.55–0.65), as compared to 0.58 (95% CI 0.53–0.63) for human expert predictions based on the same TMA samples.

**Conclusions:**

Our findings demonstrate the feasibility of learning prognostic signals in tumour tissue images without domain knowledge. Although further validation is needed, our study suggests that machine learning algorithms can extract prognostically relevant information from tumour histology complementing the currently used prognostic factors in breast cancer.

**Electronic supplementary material:**

The online version of this article (10.1007/s10549-019-05281-1) contains supplementary material, which is available to authorized users.

## Background

There is a growing interest around the potential of machine learning to improve the accuracy of medical diagnostics [1]. Novel machine learning techniques have not only advanced the state-of-the-art in several pattern recognition tasks [2, 3], but also have the potential to extract clinically relevant information from complex medical imaging data sets. Especially, methods using deep learning have been successful in various medical image analysis tasks [4, 5], some of them reaching performance of experienced specialists in individual diagnostic tasks [6, 7].

Within pathology, whole-slide scanners have enabled accurate digitisation of histological samples with sub-micrometre resolution allowing for computerised analysis of the specimens with machine learning algorithms [8]. Computerised methods for detection of mitoses [9–11], infiltrating immune cells [12, 13] and other tissue entities such as segmentation of epithelial and stromal tissue compartments or discrimination between viable and non-viable tissue [14–18] have been studied. Recent reviews [5, 8, 19] offer thorough summaries on methods developed for analysis of histological samples.

Specific type of deep learning methods, convolutional neural networks (CNNs), is frequently used in the development of the image-based classifiers. CNNs are composed of consecutive, interconnected and hierarchical stages—an architecture inspired by the structure of biological neural networks [20]—making it a powerful tool to capture abstract patterns in visual data. Utility of CNNs was recently demonstrated in detection of breast cancer metastases in lymph node tissue sections [21].

Instead of predicting patient outcome based on intermediate quantification of tissue structures, such as specific cell types and states (e.g. mitotic cells, pleomorphic cells, immune cells) or tissue structures and entities (ducts, necrosis, vessels), our aim in this study is to predict patient outcome based solely on the visual appearance of the breast cancer tissue without any prior assumptions. We hypothesise that a method capable of inferring relevant signals for outcome without prior knowledge of tissue structures may be able to reveal complementary and unbiased prognostic information.

## Materials and methods

### Patients and preparation of tumour tissue microarrays

We pooled two data sets for the study, the FinProg series and a similar single-centre series from Helsinki University Central Hospital. The FinProg series (*N* = 1860) is a nationwide cohort including approximately 50% of all women diagnosed with breast cancer in Finland 1991 and 1992 [22] and cover most of the patients (93%) within five selected geographical regions (the FinProg Breast Cancer Database^1^). The other patient series (*N* = 527) consists of patients diagnosed mainly in the Helsinki region and treated at the Department of Surgery and Oncology, Helsinki University Hospital, from 1987 to 1990. Both series include comprehensive information on clinical and pathologic characteristics extracted from the hospital and laboratory records. In the current study, we used information on histological grade and type, tumour size, number of positive lymph nodes, patient age, as well as oestrogen (ER), progesterone (PR) and human epidermal growth factor receptor 2 (HER2) status. In addition, we had information on treatment type given; 62% of the patient received local therapy and 42% systemic therapy.

Routinely fixed paraffin-embedded breast cancer samples were retrieved from the archives of pathology laboratories, and representative tumour regions identified for preparation of TMA blocks [23]. From the tissue blocks available, three representative 0.60 mm tissue cores were punched and assembled into 23 TMA blocks, each containing 50–144 tumour tissue cores. Immunohistochemistry, chromogen in situ hybridisation, as well as grading [24] were performed as previously described [22].

Inclusion criteria for the current study were the following: survival data with cause of death, images of the breast tumour tissue, as well as a tissue sample area > 0.02 mm^2^ (corresponding to 400,000 pixels in the image). Patients with lobular or ductal carcinoma in situ, synchronous or metachronous bilateral breast cancer or other malignancy (except for basal cell carcinoma or cervical carcinoma in situ), distant metastasis, or who did not undergo surgery of the primary tumour were excluded. In addition, the TMAs that were checked for quality and non-representative, samples without tumour tissue, were excluded. After exclusions, 1299 tissue spots, one per patient, were available for further analysis. Lastly, the spots were randomly divided into separate training (*N* = 868, 67%) and test (*N* = 431, 33%) sets. Compared to the commonly used 80–20% split, we assigned more samples (33%) to the test set in order to also enable subgroup and multivariate analyses. The median follow-up of patients in the final patient cohort alive at the end of follow-up period is 15.9 years (range 15.0–20.9, interquartile range 15.4–16.3 years).

### Image acquisition

Five-micrometre thick sections were cut from the TMA blocks, stained with haematoxylin and eosin and digitised with a whole-slide scanner (Pannoramic 250 FLASH, 3DHISTECH Ltd., Budapest, Hungary) equipped with a 20 × objective (numerical aperture 0.80) and a 1 × adapter, and a progressive scan colour camera with three separate charge-coupled devices with 1 618 × 1 236 pixels sized 4.40 μm × 4.40 μm (CIS_VCC_F52U25CL, CIS Corporation, Tokyo, Japan) resulting in an image where one pixel represents an area of 0.22 μm × 0.22 μm. Images were stored in a whole-slide image format (MRX, 3DHISTECH Ltd., Budapest, Hungary) and further compressed to a wavelet file format (Enhanced Compressed Wavelet, ECW, ER Mapper, Intergraph, Atlanta, GA) with a compression ratio of 1:10. The compressed virtual slides were uploaded to a whole-slide image management server (WebMicroscope, Aiforia Technologies Oy, Helsinki, Finland) where individual images of TMA spots were segmented from the whole-slide image and downloaded for algorithm training and testing as uncompressed portable network graphics files.

### Outcome classification

We extracted local convolutional image descriptors for each TMA spot image by reading the activations from the last convolutional layer of convolutional neural network (VGG-16) trained on the ImageNet database [25], and used improved Fisher vector (IFV) encoding [26] to aggregate the descriptors from the image foreground regions into a single descriptor. The network used (VGG-16) is a 16-layer network with small 3 × 3 convolutional filters. The network was not trained or fine-tuned on our data set, instead we used it only as a feature extractor. A benefit of the descriptor aggregation approach is that an image of arbitrary size can be given as an input for the model. In addition, a study [27] showed that descriptor aggregation with IFV might yield stronger discrimination when compared to fully connected layers. For computation of the IFV, the convolutional image descriptors were compressed with principal component analysis (PCA) from 512 channels into 16 components, and 64 mixture components were used in quantising the data with a Gaussian mixture model (GMM). We defined the image foreground regions by applying Otsu’s thresholding [28] to a transformed spot image $I_{t}$, when $I_{t} = (\ln \left( {1 - I_{g} } \right) + 2e) / 2e$, and where $I_{g}$ is a Gaussian-filtered (radius of 15 pixels) grayscale version (averaged over colour channels) of the spot image. After the thresholding, all objects smaller than 12,500 pixels in area were removed. Finally, we compressed the IFV descriptors with PCA into 48 bins before classification with a linear support vector machine (SVM). The analysis pipeline was implemented in a numerical computing environment (MATLAB R2016b, MathWorks, Natick, MA, U.S.) using libraries for computer vision and machine learning [29–31].

For training the DRS group classifier, we defined two categories according to the patients’ survival status and follow-up time. In the first category (*high risk*, *N* = 340), we included all the patients who died due to breast cancer earlier than 10 years after the diagnosis, and in the other category (*low risk*, *N* = 528), we included the patients who did not die of breast cancer during the entire follow-up time. For learning the unsupervised IFV encoding, we randomly sub-sampled 4 × 10^6^ local image descriptors from the training set. The sampling was balanced between low-risk and high-risk spots. In training the SVM model, we used 868 breast cancer TMA spot images, each spot representing an individual patient.

### Visual risk scoring

Three pathologists scored the test set TMA spot images into low and high-risk groups using a web-based viewing and annotation software (WebMicroscope, Aiforia Technologies Oy, Helsinki, Finland). Prior and during the visual scoring, the pathologists were able to view the training set TMA spots grouped as they were labelled in training of the SVM classifier. Based on the pathologists’ scoring, a visual risk score (high risk or low risk) was formed with majority voting. Furthermore, one pathologist assessed the following tissue entities in each TMA spot: mitoses (0 vs. 1 vs. > 1), pleomorphism (minimal vs. moderate vs. marked), tubules (≤ 10 vs. 10–75 vs. > 75%), necrosis (absent vs. present) and quantity of tumour-infiltrating lymphocytes (TILs) (low vs. high).

### Statistical analysis

The Kaplan–Meier method was used for estimating the survival function [32] and the log-rank test was used in comparison of survival curves. The disease-specific survival (DSS) time was defined as the time period between date of diagnosis and death of breast cancer, censoring patients who were alive on the date of the last contact, and those who had died from another cause on the date of death. The overall survival (OS) time was defined as the time period between the date of breast cancer diagnosis and death of any cause, censoring patients alive on the date of the last contact. For estimating the effect size (hazard ratio, HR) while accounting for the effect of other covariates, we used the Cox proportional hazard model [33]. C-index (concordance) and AUC were used to evaluate the discrimination and prediction accuracy of survival models [34]. Chi-squared contingency table test was used for comparison of categorical variables, and continuous distributions were compared with Kruskal–Wallis test. All statistical analyses with a two-sided *p* value lower than 0.05 were considered significant.

## Results

### Outcome classification

**Fig. 1:**
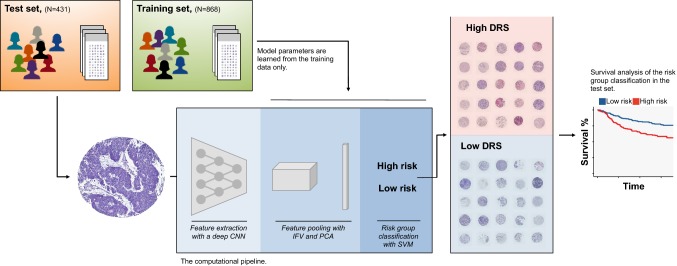
Workflow for training and testing the digital risk score (DRS) classification. The computational pipeline consists of three sequential steps: (i) feature extraction with a deep convolutional neural network (CNN), (ii) feature pooling with improved Fisher vector encoding (IFV) and principal component analysis (PCA) and (iii) classification with support vector machine (SVM). Training set samples are used in supervision and a separate test set-up used in validation

We trained the outcome classifier using a training set of 868 tumour tissue images, and subsequently classified the test set representing 431 breast cancer patients into low and high DRS groups (Fig. 1). In the test set, 237 (55%) patients were classified into the low DRS group and 194 (45%) patients into the high DRS group. The patient characteristics are summarised in Table 1. The DRS model performance rates measured with area under receiver operating characteristics curve (AUC) on the test and training sets were 0.58 and 0.63, respectively, indicating no substantial model overfitting (Supplementary Fig. S1).

**Table 1:** Patient characteristics

Variables	Whole data set (*N* = 1299)					Test set (*N* = 431)				
	Training set (*N* = 868)		Training set (*N* = 431)		*p* Value	Low DRS (*N* = 237)		High DRS (*N* = 194)		*p* Value
	%	*N*	%	*N*		%	*N*	%	*N*	
Number of positive lymph nodes										
Mean	1.4		1.2		0.407	0.9		1.6		**0.003**
0	58	504	59	253	0.323	63	150	53	103	0.057
1–3	24	206	23	99		23	54	23	45	
4–9	8	73	9	38		6	15	12	23	
> 10	3	30	2	7		1	2	3	5	
Unknown	6	55	8	34		7	16	9	18	
Tumour size (per mm)										
Mean	23.7		23.2		0.817	2.15		25.3		**< 0.001**
Unknown	3	28	5	22		5	13	5	9	
Histological grade										
I	16	143	19	83	0.086	23	54	22	43	**0.014**
II	34	296	36	154		32	75	41	79	
III	23	197	18	76		14	33	22	43	
Unknown	27	232	27	118		32	75	22	43	
Histological type										
Ductal	76	662	77	333	0.742	74	175	81	158	0.079
Lobular/special	24	206	23	98		26	62	19	36	
Age										
≤ 39	7	63	7	30	0.353	9	21	5	9	0.140
40–49	21	186	24	103		27	64	20	39	
50–59	27	234	22	94		21	49	23	45	
60–69	20	172	21	91		20	47	23	44	
≥ 70	25	213	26	113		24	56	29	57	
ER										
Negative	29	248	27	116	0.572	25	60	29	56	0.443
Positive	62	538	64	274		65	155	61	119	
Unknown	9	82	10	41		9	22	10	19	
PR										
Negative	42	362	41	177	0.803	36	86	47	91	**0.015**
Positive	49	423	50	215		56	132	43	83	
Unknown	10	83	9	39		8	19	10	20	
HER2										
Negative	72	623	74	321	0.713	76	181	72	140	0.136
Positive	17	146	16	70		14	32	20	38	
Unknown	11	99	9	40		10	24	8	16	

*Left* association of clinicopathological variables in the training and test sets. *Right:* association of clinicopathological variables between patients in low and high digital risk score (DRS) groups

*p*-values < 0.05 are shown in bold

### Outcome classification and clinicopathological variables

Patients predicted to have an increased risk of breast cancer-specific death had significantly greater proportion of high-grade tumours (*p* = 0.014) as compared to patients who were assigned to the low DRS group (Table 1). Moreover, patients in the high DRS group had larger tumours (*p* < 0.001), higher number of positive lymph nodes (*p* = 0.003) and were more often PR-negative (*p* = 0.015).

### Outcome classification and survival analysis

**Fig. 2:**
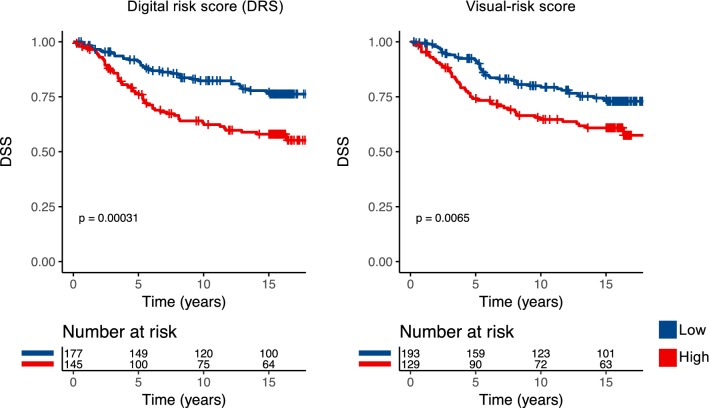
Disease-specific survival (DSS) and overall survival (OS) according the classification into low and high digital risk score (DRS) groups

**Fig. 3:**
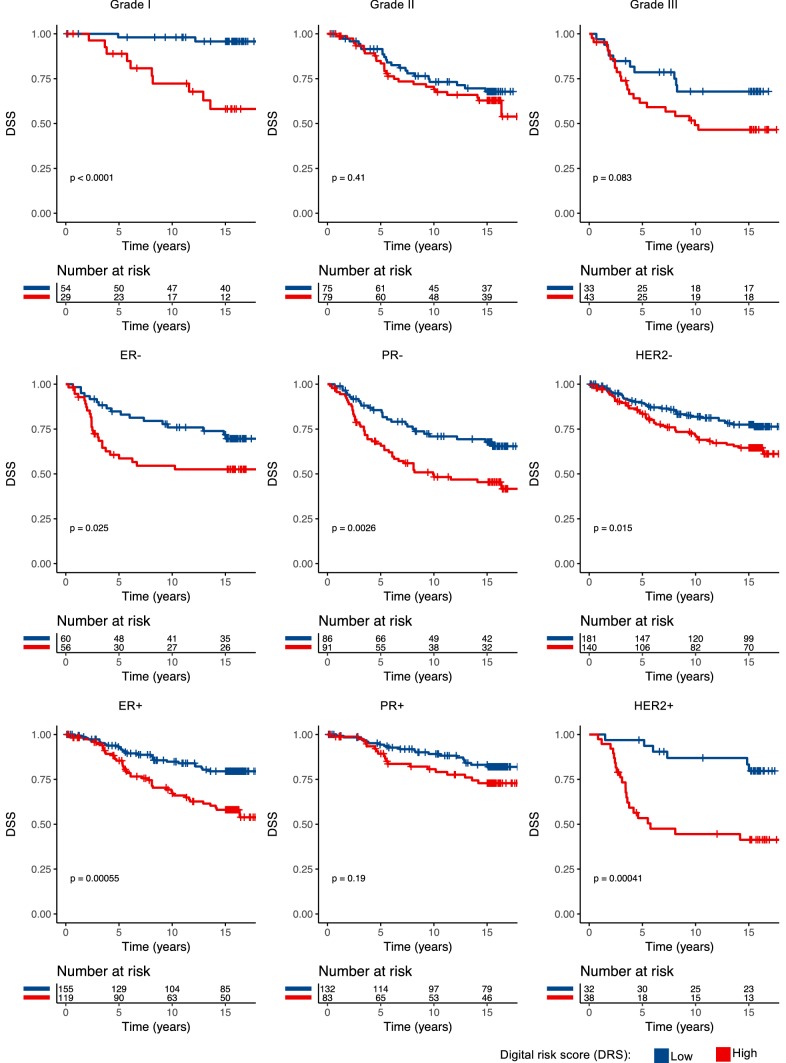
Disease-specific survival (DSS) according the classification into low and high digital risk score (DRS) groups

**Fig. 4:**
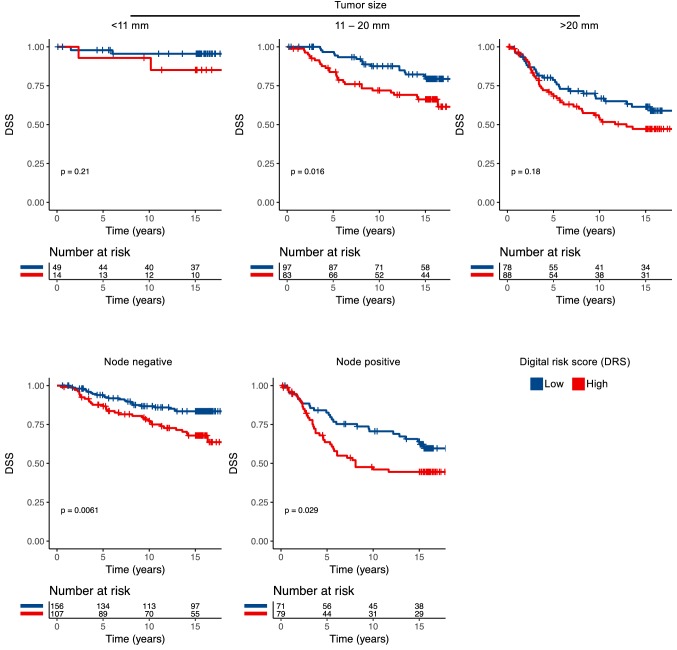
Disease-specific survival (DSS) according the classification into low and high digital risk group (DRS) groups in patients with different tumour size and nodal status

We investigated the prognostic value of the DRS grouping with univariable and multivariable survival analysis in the test set. Women in the lower DRS group had more favourable breast cancer-specific (*p* < 0.001) and overall survival (*p* = 0.003) (Fig. 2). Ten-year DSS in the low DRS group was 82% (95% CI 77–87%) compared to 65% (95% CI 58–73%) in the high DRS group. When the cancers were split according to histological grade assessed from original whole-slide samples, the DRS grouping showed the strongest discrimination in grade I cancer (P < 0.001), whereas the differences observed in grade II (*p* = 0.410) and grade III (*p* = 0.083) groups were not statistically significant (Fig. 3). When the cancers were divided according the steroid hormone receptor status, the DRS classifier was a significant predictor of survival both in the ER positive (*p* = 0.025) and ER negative (*p* < 0.001) subgroups. The DRS grouping was a significant predictor in the PR-negative subgroups, but not in the subset of PR-positive breast cancer (*p* = 0.003). Furthermore, the risk grouping was a significant predictor for survival both among HER2 negative (*p* = 0.015) and positive patients (*p* < 0.001). Subgroup analysis according to tumour size and nodal status are shown in Figure 4.

A multivariate survival analysis showed that classification into the high DRS group was associated with unfavourable prognosis (HR = 2.04, 95% CI 1.20–3.44, *p* = 0.007), and indicated that the DRS grouping is an independent predictor for survival (Table 2). Tumour size (1.04, 95% CI 1.02–1.06, *p* < 0.001), PR positivity (0.42, 95% CI 0.25–0.71, *p* < 0.001) and having > 10 positive lymph nodes (HR = 4.74, 95% CI 1.17–19.30, *p* < 0.029) were also independent predictors.

**Table 2:** Cox uni- and multivariate survival analysis

Variables	Univariate analysis			Multivariate analysis		
	HR	CI 95%	*p* value	HR	CI 95%	*p* Value
DRS						
Low	Ref.			Ref.		
High	2.10	(1.33–3.32)	**0.001**	2.04	(1.20–3.44)	**0.007**
Number of positive lymph nodes						
0	Ref.			Ref.		
1–3	1.53	(0.89—2.63)	0.123	2.12	(0.83–1.47)	0.116
4–9	2.93	(1.61–5.33)	**<** **0.001**	2.15	(0.75–6.19)	0.154
> 10	7.43	(2.90–19.02)	**<** **0.001**	4.75	(1.17–19.30)	**0.029**
Tumour size						
Per mm	1.04	(1.03–1.06)	**<0.001**	1.04	(1.02–1.06)	**<0.001**
Histological grade						
I	Ref.			Ref.		
II or III	3.14	(1.61–6.09)	**<** **0.001**	1.57	(0.76–3.20)	0.220
Histological type						
Ductal	Ref			Ref.		
Lobular/special	0.73	(0.40–1.33)	0.306	0.90	(0.41–1.95)	0.782
Age						
≤ 39	Ref.			Ref.		
40–49	0.78	(0.33–1.88)	0.585	0.43	(0.17–1.12)	0.084
50–59	0.69	(0.28–1.70)	0.425	0.48	(0.19–1.28)	0.144
60–69	1.00	(0.42–2.36)	0.996	0.62	(0.25–1.57)	0.319
≥ 70	1.58	(0.66–3.79)	0.306	1.35	(0.49–3.72)	0.564
ER						
Negative	Ref.					
Positive	0.69	(0.44–1.09)	0.15			
PR						
Negative	Ref.			Ref.		
Positive	0.34	(0.21–0.55)	**< 0.001**	0.42	(0.25–0.71)	**0.001**
HER2						
Negative	Ref.					
Positive	1.51	(0.90–2.53)	0.119	1.07	(0.57–1.98)	0.831
Systematic therapy						
Not given	Ref.			Ref.		
Given	1.90	(1.21–2.98)	**0.005**	1.07	(0.22–1.47)	0.245
Local therapy						
Not given	Ref.			Ref		
Given	1.23	(0.75–2.03)	0.404	1.22	(0.61–2.46)	0.571

Histological grade and type were assessed from whole tumour sections, while ER, PR and HER2 were assessed from TMAs. In order to meet the Cox proportionality assumption, ER was left out from the multivariate analysis and grade II and III were combined

*ER* estrogen receptor status, *PR* progesterone receptor status, *HER2* human epidermal growth factor receptor 2 gene amplification

*p*-values < 0.05 are shown in bold

### Outcome classification and visual risk score

Out of the 431 test TMA spot images, 109 were classified by at least one pathologist as not evaluable due to insufficient amount of cancer tissue or partial spot detachment for reliable risk assessment and were therefore left out from the analyses.

In the remaining subset of 322 spot images, 60% (*N* = 193) of the patients were assigned to the low-risk and 40% (*N* = 129) to the high-risk group according the majority vote visual risk score, as compared to 55% (*N* = 177) low risk and 45% (*N* = 145) high risk according to the DRS groups. Percent agreement between the pathologists’ individual scorings was 32%. There was a significant agreement between pathologist 1 and 3 (*κ*_(1,3)_ = 0.27; *p* < 0.001), but not between pathologist 1 and 2 (*κ*_(1,2)_ = 0.005; *p* = 0.931) or pathologist 2 and 3 (κ_(2,3)_ = − 0.028; *p* = 0.598) in assigning the patients into low- and high-risk groups.

In a univariate analysis, the digital risk score was found to be a significant predictor of disease-specific survival with a HR = 2.10 (95% CI 1.40–3.18, *p* < 0.001) and C-index of 0.60 (95% CI 0.55–0.65). Similarly, the visual risk score was found to be a significant predictor of survival with a HR = 1.74 (95% CI 1.16–2.61, *p* = 0.006) and C-index of 0.58 (95% CI 0.53–0.63) (Supplementary Fig. 2). Interestingly, a Chi-square test of these univariate survival models indicated that the models were significantly different (*p* < 0.001). When the visual risk score and the DRS group were both included as covariables in a multivariate survival analysis, both turned out to be independent predictors (HR = 2.05, *p* < 0.001 for the DRS and HR = 1.68, *p* = 0.012 for the visual risk score). C-index of the combined survival model was 0.64 (95% CI 0.58–0.69). An analysis of the association between cancer morphological features and the digital risk score showed that the DRS was significantly correlated with nuclear pleomorphism and tissue tubule formation, whereas the visual risk score was significantly associated also with cancer mitotic count, presence of necrosis and the number of TILs (Supplementary Table 1).

## Discussion

We found that by utilising machine learning algorithms it is possible to extract information relevant for breast cancer patient outcomes from tumour tissue images stained for the basic morphology only. Importantly, the results show that prognostic discrimination is achievable without guidance or the use of prior knowledge of breast cancer biology or pathology in the training of the algorithm. Instead of directing the focus towards cancer cellular features (e.g. number of mitotic cells, immune cells, pleomorphic cells) or tissue entities (e.g. duct formation, presence of tumour necrosis), we guided the supervision simply with the patient survival outcome data.

Computerised methods for analysing breast tissue images for patient prognostication have been studied earlier. Extracting more than 6000 predefined image features from two cohorts (*N* = 248 and *N* = 328), authors in [35] showed that it is feasible to learn an outcome predictor for overall survival (HR = 1.78, *p* = 0.017) using small tumour regions. Using a subset of the whole slides from [36], an earlier study [37] proposed a joint analysis of image features and gene expression signatures for prognostic biomarker discovery. The authors used a training set of 131 patients and validated the biomarkers with H&E-stained tumour samples from 65 breast cancer patients. The strongest predictive image feature the authors identified reached a HR = 1.7 (*p* = 0.002) in prediction of relapse-free survival. Moreover, a previous work [38] identified morphological features in a data set of 230 breast cancer patients that were independent and prognostic for 8-year disease-free survival. Our study extends this body of work by demonstrating that it is possible to learn a prognostic signal from a patient cohort without domain knowledge. Our analysis was blinded from the fundamental concepts such as cells and nuclei, different tissue compartments and histological grade that were incorporated in the previous studies. Nevertheless, we were able to train an independent risk predictor based on the training cohort, using the raw image data and follow-up information only. Furthermore, we used a large multicentre cohort with a median follow-up time of over 15 years and analysed the associations of the outcome predictor with the commonly used clinicopathological variables. Outside breast cancer, direct outcome prediction using tissue morphology has been successfully applied in colorectal cancer [39] and glioma [40].

Moreover, we compared the DRS group with the visual risk score, which combined three pathologists’ risk assessments according to a majority vote rule. Even though pathologists do not perform such a direct risk assessment as part of breast cancer diagnostics, we wanted to evaluate the prognostic potential of morphological features detected by pathologists in a small tumour tissue area (a TMA core) and compare this with the corresponding digital risk score. The analysis indicated that the visual risk score was a significant predictor of outcome, but that the digital risk score yielded a slightly stronger discrimination than the visual risk score (C-index 0.60 vs. 0.58). As expected, the visual risk score correlated with known tissue entities (mitoses, pleomorphism, tubules, necrosis and TILs). Interestingly, the DRS group associated only with pleomorphism and tubules, indicating that the machine learning algorithm partly has learned known prognostic entities, but partly has extracted features that are not fully explained by known factors. This was supported by multivariate survival analysis with DRS and visual risk score, which showed increased discrimination (C-index 0.64), and revealed that the risk scores are independent prognostic factors.

One of the main reasons behind the success of deep learning and CNNs has been improved availability of large data sets [41, 42]. The best-performing CNNs for object detection and classification are trained with millions of images [43–45]. Contrary to classification based on handcrafted image descriptors and shallow learners, CNN inherently learns hierarchical image features from data, and larger data set usually leads into more powerful models. This ability to learn features directly from the data makes CNNs perform well and why they are easy to use. However, when only limited number of data points is available, direct end-to-end training of a CNN might not lead into any added benefit over handcrafted features and a shallow classifier.

Our goal in the design of the computational pipeline for patient outcome classification was to combine the best from the both worlds; the descriptive power of CNNs with the capability of shallow learners to generate robust models from more limited data set. Generally, this approach is known as transfer learning, which is a popular strategy to achieve a strong performance even with smaller data sets [46, 47]. We took advantage of a CNN trained on the ImageNet [48], a large database for object recognition, and used it for extracting local image descriptors. An important benefit of this approach is less computational requirements, since training of the CNN is not needed. Furthermore, the approach is agnostic with regard to the CNN used and is easily amendable and compatible with novel model architectures frequently discovered and shared online for the research community. The ImageNet consists of photographs representing natural objects from bicycles to goldfish.^2^ Histological images are fundamentally different from everyday photos and it is reasonable to assume that the descriptors learned in natural images are not optimally suited for analysis of tumour tissue images. IFV is an orderless descriptor aggregation method, capturing the first- and second-order statistics of the GMM modes. The GMM modes were learned in the training set of tumour tissue images, and therefore this intermediate unsupervised learning phase further fine-tunes the features more suitable to the domain of histological images.

Our study has some important limitations. The cohort used in this study was centrally scanned using the same slide scanner and therefore the generalisation of the outcome prediction to tissue images from other slide scanners was not taken into consideration. Moreover, our study considered only small tumour area in the form of a TMA spot image.

Although our analysis indicated correlation with the computerised prediction and pleomorphism and tubules, a major limitation of the current work is the difficulty to explain the exact source and location of the predictive signal, i.e. which tissue regions gave rise to the result obtained. Deep learning models are considered as “black boxes”, which work well, but whose function, or reasoning, is difficult to reveal [49]. Some approaches to answer this shortcoming have been presented [50], but this is an active research question in field of machine learning and no direct solution for this exists at present. We intend to address this in the future studies.

Our findings indicate that computerised methods offer an innovative approach for analysing histological samples. Nevertheless, future studies are required to validate our findings, test similar algorithms on larger data sets representing different malignancies.

## Conclusions

We have demonstrated how machine learning analysis of tumour tissue images can be utilised for breast cancer patient prognostication. Our results show that it is possible to learn a risk grouping, providing independent prognostic value complementing the conventional clinicopathological variables, using only digitised tumour tissue images and patient outcome as the endpoint. These findings suggest that machine learning algorithms together with large-scale tumour tissue image series may help approximate the full prognostic potential of tumour morphology.

## Electronic supplementary material

Below is the link to the electronic supplementary material.

Supplementary material 1 (PDF 96 kb)

## Data Availability

The data that support the findings of this study are available from the University of Helsinki but restrictions apply to the availability of these data, which were used under license for the current study, and so are not publicly available. Data are however available from the authors upon reasonable request and with permission of University of Helsinki.

## Abbreviations

AUC: Area under receiver operating characteristics curve
CI: Confidence interval
CNN: Convolutional neural network
DSS: Disease-specific survival
DRS: Digital risk score
ECW: Enhanced wavelet compression
ER: Oestrogen receptor
GMM: Gaussian mixture model
HER2: Human epidermal growth factor receptor 2
HR: Hazard ratio
IFV: Improved Fisher vector
OS: Overall survival
PCA: Principal component analysis
PR: Progesterone receptor
SVM: Support vector machine
TIL: Tumour-infiltrating lymphocytes
TMA: Tissue microarray

## Footnotes

1 http://www.finprog.org.

2 http://www.image-net.org/.
