# Supplementary material for: Breast cancer outcome prediction with tumour tissue images and machine learning
Source: Breast Cancer Res Treat. 2019 May 22;177(1):41–52. doi: 10.1007/s10549-019-05281-1 (PMC6647903; doi:10.1007/s10549-019-05281-1)
Supplement: Supplementary file 1 — Supplementary material 1 (PDF 96 kb) [file 10549_2019_5281_MOESM1_ESM.pdf]

Supplementary material

Breast cancer outcome prediction with tumour tissue images and machine learning

Riku Turkki, Dmitrii Byckhov, Mikael Lundin, Jorma Isola, Stig Nordling, Panu E Kovanen, Clare Verrill, Karl von Smitten, Heikki Joensuu, Johan Lundin, Nina Linder

Corresponding author: Riku Turkki, riku.turkki@helsinki.fi

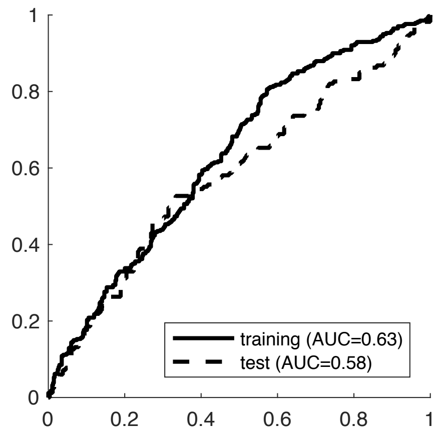

Additional figure 1. Model performance on training and test data.

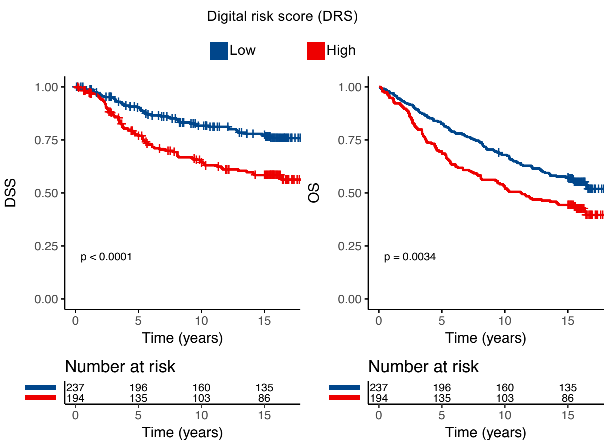

Additional figure 2. Disease specific survival (DSS) and overall survival (OS) according digital risk (DRS) group and visual-risk score.

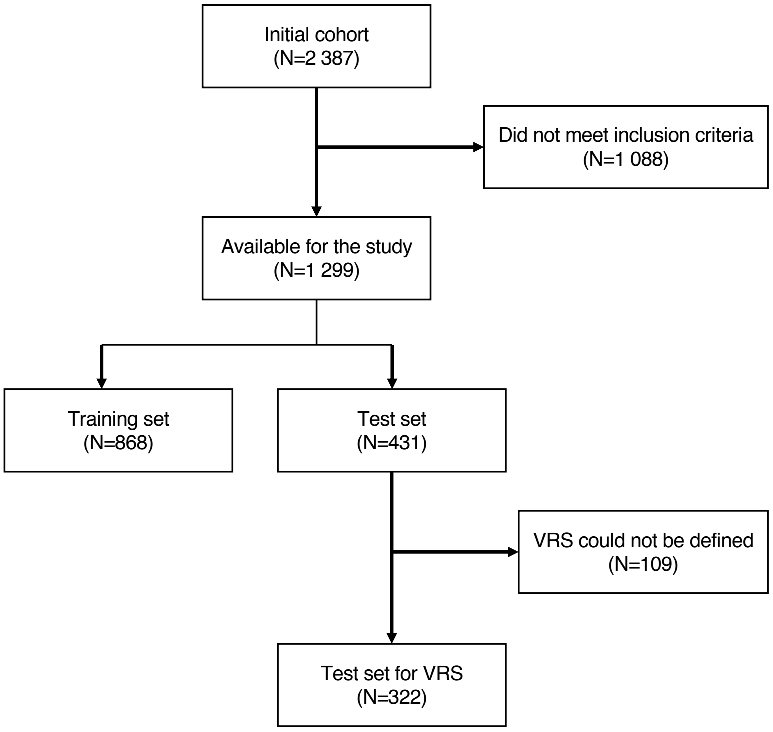

Additional figure 3. CONSORT flow diagram.

Variables	Digital risk score Low (N=177)		High (N=145)			Visual risk score Low (N=193)		High (N=129)		
	%	N	%	N	P-value	%	N	%	N	P-value
**Mitoses**										
0	84 %	148	74 %	107		96 %	186	53 %	69	
1	9 %	16	15 %	22	0.095	2 %	3	27 %	35	**<0.001**
>1	7 %	13	11 %	16		2 %	4	19 %	25	
**Pleomorphism**										
Minimal	22 %	39	6 %	8		21 %	41	5 %	6	
Moderate	59 %	105	60 %	87	**<0.001**	72 %	139	41 %	53	**<0.001**
Marked	19 %	33	34 %	50		7 %	13	54 %	70	
**Tubules, %**										
<10	81 %	144	88 %	127		76 %	146	97 %	125	
10-75	14 %	24	12 %	18	**0.020**	20 %	38	3 %	4	**<0.001**
>75	5 %	9	0 %	0		5 %	9	0 %	0	
**Necrosis**										
Absent	95 %	169	98 %	142	0.370	99 %	192	92 %	119	**0.001**
Present	5 %	8	2 %	3		1 %	1	8 %	10	
**TILs**										
Low	90 %	160	83 %	120	0.063	91 %	175	81 %	105	**0.024**
High	10 %	17	17 %	25		9 %	18	19 %	24	

Additional table 1. Association with tumour tissue entities. Left: association of tissue entities between patients in low and high digital risk score (DRS) groups. Right: association of tissue entities between patients in low and high by visual-risk score.
